# Plasma metabolomic differences in early-onset compared to average-onset colorectal cancer

**DOI:** 10.1038/s41598-024-54560-5

**Published:** 2024-02-21

**Authors:** Thejus Jayakrishnan, Arshiya Mariam, Nicole Farha, Daniel M. Rotroff, Federico Aucejo, Shimoli V. Barot, Madison Conces, Kanika G. Nair, Smitha S. Krishnamurthi, Stephanie L. Schmit, David Liska, Alok A. Khorana, Suneel D. Kamath

**Affiliations:** 1 Department of Hematology and Oncology, Taussig Cancer Institute, Cleveland Clinic, Cleveland, USA; 2 Department of Quantitative Health Sciences, Lerner Research Institute, Cleveland Clinic, Cleveland, USA; 3 Center for Quantitative Metabolic Research, Cleveland Clinic, Cleveland, USA; 4 Department of Surgery, Digestive Disease & Surgery Institute, Cleveland Clinic, Cleveland, USA; 5 Case Comprehensive Cancer Center, Cleveland, USA; 6 Department of Hematology-Oncology, University Hospital Seidman Cancer Center, Cleveland, USA; 7 Center for Young-Onset Colorectal Cancer, Cleveland Clinic, Cleveland, USA; 8 Genomic Medicine Institute, Lerner Research Institute, Cleveland Clinic, Cleveland, USA; 9 Population and Cancer Prevention Program, Case Comprehensive Cancer Center, Cleveland, USA; 10 Department of Colorectal Surgery, Digestive Disease & Surgery Institute, Cleveland Clinic, Cleveland, USA; 11 Cleveland Clinic Lerner College of Medicine of Case Western Reserve University, Cleveland, OH USA

**Keywords:** Early onset colorectal cancer, Metabolomics, Pathway analysis, Citric acid cycle, Arginine biosynthesis pathway, Synthetic lethality, Translational research, Translational research, Colorectal cancer

## Abstract

Deleterious effects of environmental exposures may contribute to the rising incidence of early-onset colorectal cancer (eoCRC). We assessed the metabolomic differences between patients with eoCRC, average-onset CRC (aoCRC), and non-CRC controls, to understand pathogenic mechanisms. Patients with stage I–IV CRC and non-CRC controls were categorized based on age ≤ 50 years (eoCRC or young non-CRC controls) or  ≥ 60 years (aoCRC or older non-CRC controls). Differential metabolite abundance and metabolic pathway analyses were performed on plasma samples. Multivariate Cox proportional hazards modeling was used for survival analyses. All *P* values were adjusted for multiple testing (false discovery rate, FDR *P* < 0.15 considered significant). The study population comprised 170 patients with CRC (66 eoCRC and 104 aoCRC) and 49 non-CRC controls (34 young and 15 older). Citrate was differentially abundant in aoCRC vs. eoCRC in adjusted analysis (Odds Ratio = 21.8, FDR *P* = 0.04). Metabolic pathways altered in patients with aoCRC versus eoCRC included arginine biosynthesis, FDR *P* = 0.02; glyoxylate and dicarboxylate metabolism, FDR *P* = 0.005; citrate cycle, FDR *P* = 0.04; alanine, aspartate, and glutamate metabolism, FDR *P* = 0.01; glycine, serine, and threonine metabolism, FDR *P* = 0.14; and amino-acid t-RNA biosynthesis, FDR *P* = 0.01. 4-hydroxyhippuric acid was significantly associated with overall survival in all patients with CRC (Hazards ratio, HR = 0.4, 95% CI 0.3–0.7, FDR *P* = 0.05). We identified several unique metabolic alterations, particularly the significant differential abundance of citrate in aoCRC versus eoCRC. Arginine biosynthesis was the most enriched by the differentially altered metabolites. The findings hold promise in developing strategies for early detection and novel therapies.

## Introduction

The incidence of early-onset colorectal cancer (eoCRC), defined as CRC diagnosed at age less than 50 years, has been steadily increasing, leading to the median age of diagnosis shifting from 72 years in the early 2000s to 66 years at present^1–4^. The increasing incidence of eoCRC has recently prompted the American Cancer Society and US Preventive Services Task Force to recommend the initiation of screening earlier at age 45^2,5^. Many of the environmental risk factors for average-onset CRC (aoCRC) are also relevant for eoCRC^2^. These include lifestyle factors such as physical inactivity and high red meat consumption^6^. However, the drivers of the increasing trend, and potential novel risk factors or differential contributions of known/established risk factors, are not well understood.

Metabolomics involves global analysis of small molecule metabolites in body fluids or tissue extracts^7^. Evolving clinical utility of metabolomics such as risk prediction in cardiovascular disease has been demonstrated^8,9^. In cancer, metabolomics may reflect the alterations resulting from cancer, and can identify the pathophysiologic changes preceding cancer development related to one's exposures. Therefore, it could serve to identify cancer risk factors, study cancer biology, and develop novel therapeutics^10,27^. Studies have identified metabolic alterations associated with CRC but such data in the context of eoCRC are limited^10–12^. In the present study, we aimed to identify metabolomic differences between patients with eoCRC and aoCRC in comparison to non-CRC controls.

## Methods

### Patient samples

Patient plasma samples were obtained from prospective colorectal and liver tumor biobanks at the Cleveland Clinic from 01/2004 to 03/2021. Blood samples were obtained from patients on the day of their procedures, logged, and immediately stored at − 80 °C until processed. The prospective biobanks and studies on banked specimens were approved by the Institutional Review Board (IRB) at the Cleveland Clinic. All the patients were treated at Cleveland Clinic. None of them were participating in clinical trials involving metabolic interventions such as arginine synthesis modulators or tricarboxylic acid (TCA) cycle inhibitors at the time of the study.

The samples were divided into cases and non-CRC controls. The cases included patients with stages I–IV CRC. For patients with early-stage cancers (stages I–III), the samples were obtained at the time of surgical resection of the primary disease, and for the stage IV group, the samples were obtained during liver metastasis resection. The cases were excluded if they were nonmalignant, or non-adenocarcinoma. The non-CRC controls included those who underwent liver resections or biopsies for benign causes or liver transplant donors and were selected based on the availability of bio-banked plasma samples for comparison. Of note, individuals with CRC who underwent surgical resection received colon cleansing preparation. Since the stage of cancer (and therefore the type of surgery including the need for cleansing preparation) was incorporated as a covariate in the modeling, any resulting impact would be accounted for, in the present analysis. Additionally, the use of plasma instead of tissue may mitigate any effect that colon preparation might have on the metabolome^13^.

The samples were categorized based on the age at the time of sample collection as ≤ 50 years (patients with eoCRC or young non-CRC controls) or ≥ 60 years (patients with CRC or older non-CRC controls). Retrospective chart reviews of the included patients were conducted to obtain clinical information.

### Metabolomic analyses

Samples were submitted for metabolomic analyses by gas chromatography time-of-flight mass spectrometry (GC–TOF–MS) using the Primary Metabolism panel from West Coast Metabolomics at UC Davis^14^. This is a non-targeted plasma GC-TOF mass spectrometry assay of approximately 200 known and > 200 unknown metabolites including amino acid, carbohydrate and fatty acid metabolites. The list of known metabolites included in the analyses is available at https://metabolomics.ucdavis.edu/core-services/metabolites^15^.

The details of the technique including the validity of the methods, plasma extraction, and plasma metabolomics have been described previously^16–19^. To begin, the samples were subjected to extraction using a solution consisting of acetonitrile, isopropanol, and water in a ratio of 3:3:2, which was chilled to − 20 °C and degassed. A volume of 1 mL of this solution was utilized for extraction. Subsequently, 500 μL of the resulting supernatant was evaporated to dryness using a CentriVap (Labconco, Kansas, MO). For metabolite derivatization, a two-step process previously described was employed^16^. First, methoximation was employed to protect carbonyl groups, followed by the exchange of acidic protons with trimethylsilyl groups to enhance volatility. An injection of 0.5 μL sample volume was made into an Agilent 6890 GC (Agilent Technologies, Santa Clara, CA, USA), equipped with a Restek Rtx-5Sil MS column (30 m × 0.25 mm, 0.25 μm) and operated with a splitless time of 25 s and a helium gas flow rate of 1 mL/min. The oven temperature was initially held at 50 °C for 1 min and then increased to 330 °C at a rate of 20 °C/min, where it was maintained for 5 min.

Data acquisition was performed using a Leco Pegasus IV time-of-flight mass spectrometer (Leco Corporation, St. Joseph, MI) with electron ionization at − 70 eV. The mass spectra were recorded from 85 to 500 Da at a rate of 17 spectra/s and a detector voltage of 1850 V. The transfer line temperature was maintained at 280 °C, and the ion source temperature was set to 250 °C.

To ensure quality control, standard metabolite mixtures, and blank samples were injected at the beginning of the run and every ten samples throughout the analysis. Raw data were preprocessed using ChromaTOF version 4.50, which encompassed baseline subtraction, deconvolution, and peak detection. Metabolite annotation and reporting were performed using Binbase^20^. The results were further analyzed using the bioinformatics workflow described below.

### Association analyses

The associations between individual metabolites (N = 449) and disease status were investigated using logistic regression using the *stats* package in open-source statistical software R version 4.2.1 (R Foundation for Statistical Computing, Vienna, Austria)^21^. These associations were used to investigate the associations of log-normalized metabolites with (1) patients with CRC vs. non-CRC controls, (2) patients with eoCRC vs. non-CRC controls aged ≤ 50 years, (3) patients with aoCRC vs. non-CRC controls aged ≥ 60 years, (4) patients with eoCRC versus aoCRC, and (5) non-CRC controls aged ≤ 50 versus ≥ 60 years. These associations were adjusted for sex, race, hyperlipidemia, obesity, cancer stage, and primary tumor location (left-sided vs. right-sided, rectal vs. non-rectal). Results are presented as odds ratios (OR) and 95% CI. For the comparison between aoCRC and eoCRC, an OR > 1 indicated a higher abundance of metabolites associated with aoCRC. All *P* values were adjusted for multiple testing using the Benjamini–Hochberg false discovery rate approach^22^. FDR *P* < 0.15, previously used in other hypothesis-generating metabolomic studies, was used as the threshold for significance^23–25^.

### Pathway analyses

Metaboanalyst 5.0 was used for pathway analyses of the significantly altered metabolites (*P* < 0.05) using KEGG as the pathway reference. The enrichment method and topology analysis used hypergeometric tests and out-degree centralities, respectively. Pathways were considered enriched if the FDR *P* < 0.15.

### Survival analyses

Overall survival (OS) was measured in months (m) from the day of cancer diagnosis to the day of death or censoring (last follow-up). Multivariate Cox proportional hazards modeling was performed to associate any metabolite with survival in patients with CRC (eoCRC and aoCRC). The model was adjusted for relevant demographic and clinical characteristics (race, sex, hyperlipidemia, obesity, stage, tumor-sidedness, and rectal cancer status). Statistical analyses were performed using the R *survival* package version 3.5-5^26^.

### Ethical approval

This study was performed under the oversight of the Cleveland Clinic institutional review board and the ethical approval process (IRB# 4134 and IRB# 10-347).

### Conference presentation

This study was presented at the 2023 American Society of Clinical Oncology Gastrointestinal Cancers Symposium, and updated results were presented at the 2023 American Society of Clinical Oncology annual meeting as a podium presentation (Clinical cancer Symposium on the molecular basis of young-onset colorectal cancer). The first author (T.J.) received an ASCO Conquer Cancer Merit Award.

## Results

### Baseline characteristics

The study population comprised 170 patients with CRC (66 eoCRC and 104 aoCRC) and 49 non-CRC controls (34 with age < 50 years and 15 with age > 60 years). The majority of non-CRC controls were subjects with hepatic adenomas (n = 15, 31.3%), followed by healthy liver donors (n = 11, 22.9%), and those with other benign conditions of the liver, including cysts (n = 10, 20.8%), hemangiomas (n = 7, 14.6%), and focal nodular hyperplasia (n = 5, 10.4%).

The baseline characteristics of the patients are summarized in Table 1. The majority of the subjects were male (59.4%) for CRC and female (83.7%) for non-CRC controls, *P* < 0.0001. The race distribution was similar with the majority of both groups identifying as White—CRC (91.8%) and control (83.7%). The median age of patients with CRC (median = 62.95, IQR = 45.10–70.73) was greater than that of the non-CRC controls (median = 43.36, IQR = 32.83–64.35). There was no significant difference in the family history of CRC and personal or family history of colon polyps between patients with CRC and non-CRC controls. The majority had no significant family history of CRC (84.7% in CRC vs. 85.7% in control) or colon polyps (91.8 vs. 93.9%). There were no significant differences in smoking or alcohol consumption (*P* > 0.05). The differences in microsatellite instability (MSI) status and somatic mutations for patients that underwent testing are outlined in Supplementary Table 1. There was only 1 patient with MSI-H status in eoCRC (1.5%) and 4 patients (3.8%) in aoCRC.

**Table 1:** Baseline characteristics of patients selected for the analysis—n (%) for categorical variables and mean (standard deviation—SD) for continuous variables.

Characteristics	All CRC (n = 170)	eoCRC (n = 66)	aoCRC (n = 104)	*p*-value^a^	Non-CRC Control (n = 49)	*p*-value^b^
Age (SD)	59.4 (16.0)	41.8 (6.1)	70.6 (8.5)	< 0.001	47.4 (18.2)	NA
Sex: Male	101 (59.4%)	34 (51.5%)	67 (64.4%)	0.131	8 (16.3%)	< 0.001
Sex: Female	69 (40.6%)	32 (48.5%)	37 (35.6%)		41 (83.7%)	
Race: White	156 (91.8%)	62 (93.9%)	94 (90.4%)	0.592	41 (83.7%)	0.164
Race: Black	12 (7.1%)	2 (3.0%)	10 (9.6%)		7 (14.3%)	
Race: Other	2 (1.2%)	2 (3.0%)	0		1 (2.0%)	
Diabetes	35 (20.6%)	5 (7.6%)	30 (28.8%)	0.002	5 (10.2%)	0.148
Hyperlipidemia	52 (30.6%)	12 (18.2%)	40 (38.5%)	0.009	26 (53.1%)	0.006
Obesity	41 (24.1%)	22 (33.3%)	19 (18.3%)	0.04	25 (51.0%)	0.001
Sidedness: Left sided	128 (75.3%)	59 (89.4%)	69 (66.3%)	0.003	-	NA
Rectal Primary	72 (42.4%)	35 (53.0%)	37 (35.6%)	0.035	-	NA
Stage IV cancer	77 (45.3%)	41 (62.1%)	36 (34.6%)	0.001	-	NA

*p*-value ^a^For eoCRC vs aoCRC groups comparison.

*p*-value ^b^For CRC control groups comparison.

CRC, colorectal cancer; eoCRC, early-onset colorectal cancer; aoCRC, average-onset colorectal cancer; NA, not applicable.

The mean BMI for patients with CRC and non-CRC controls were 28.71 (SD = 5.81) and 30.33 (SD = 6.34), respectively. Among the comorbidities assessed, the prevalence of the following was significantly different between patients with CRC and non-CRC controls—hyperlipidemia (30.6% in CRC group vs. 53.1% in the control group, *P* = 0.006) and obesity (24.1 vs. 51.0%, *P* < 0.001), while that of diabetes mellitus (20.6 vs. 10.2%, *P* = 0.148) was similar. Inflammatory bowel disease (IBD) was reported in 4.1% of CRC patients.

A higher proportion of patients with eoCRC than with aoCRC had the left-sided disease (89.4 vs. 66.3%, *P* = 0.003), rectal primary cancer (53.0 vs. 35.6%, *P* = 0.037), and stage IV disease (62.1 vs. 34.6%, *P* = 0.001). The prevalence of IBD was similar between the groups – 6.1% among patients with eoCRC vs 2.9% among patients with aoCRC (*P* = 0.535).

Hyperlipidemia and obesity were included as covariates because the proportions of these diseases varied significantly between patients with CRC and non-CRC controls at baseline (*P* < 0.05). The complete metabolic profiles of all the patients are shown in Supplementary Fig. 1.

### Metabolomic analyses

**Figure 1:**
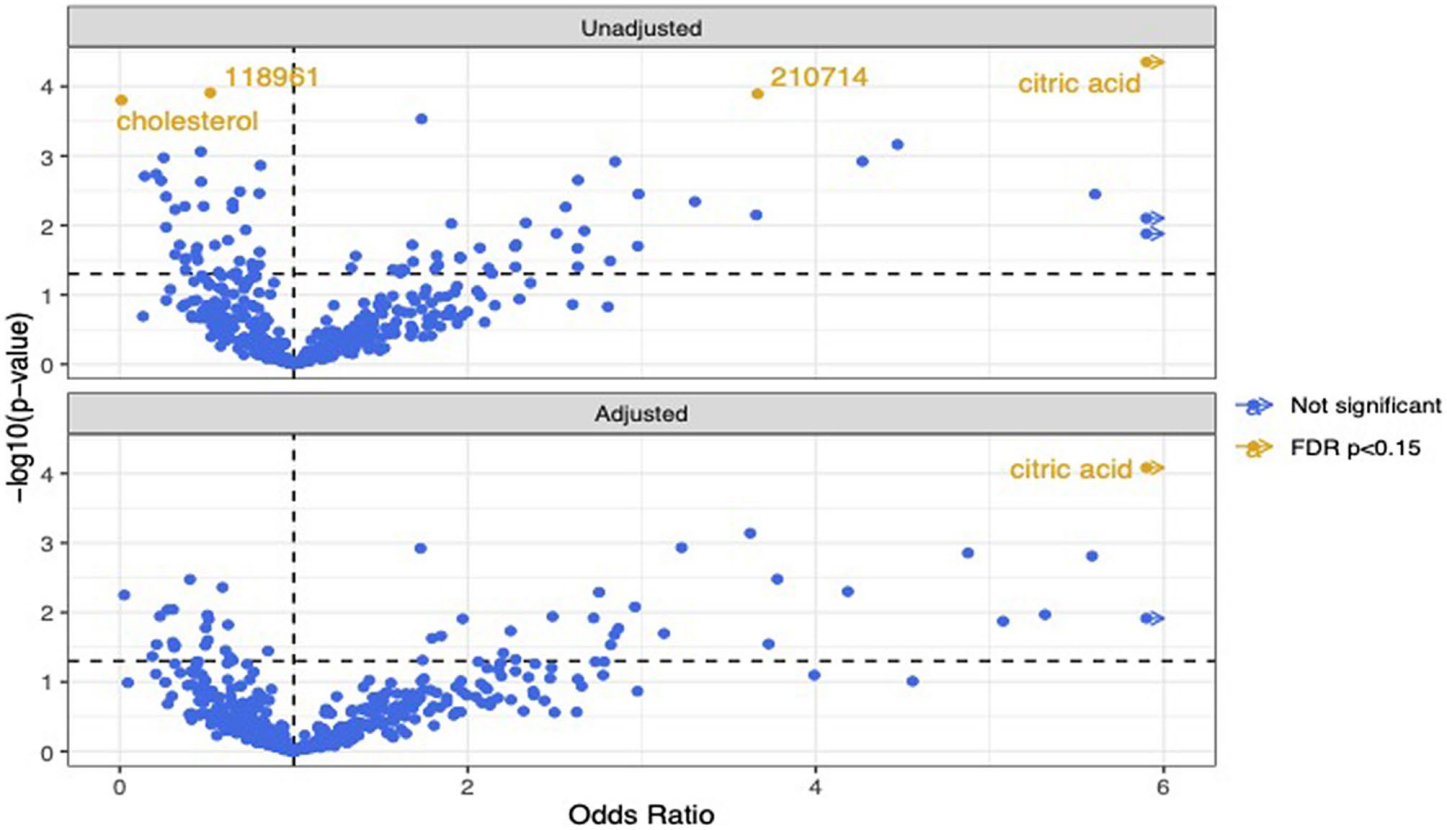
Volcano plot representing results of unadjusted and adjusted metabolomic analyses in comparing eoCRC and aoCRC. FDR, false discovery rate; corresponding values are described in Table 1.

Association analyses revealed four differentially abundant metabolites in the eoCRC versus aoCRC comparison: citrate (FDR *P* = 0.04), cholesterol (FDR *P* = 0.14), and two unidentified metabolites, UM118961 (FDR *P* = 0.11) and UM210714 (FDR *P* = 0.11), in unadjusted analyses (Fig. 1). The corresponding odds ratios were (OR > 1 indicating the association of higher abundance with aoCRC vs. eoCRC – citrate 14.54 (95% CI 4.26–56.35), cholesterol 0.01 (0.001–0.10), UM118961 0.52 (0.37–0.72), UM210714 3.67 (1.93–7.33) (Table 2). The differential abundance in citrate level remained significant on adjusted analysis—OR = 21.8 (95% CI 5.0–110.9, FDR *P* = 0.04). (Fig. 1).

**Table 2:** Results of metabolomic analyses assessing differential abundance of metabolites between patients with aoCRC versus eoCRC and pathways impacted.

Differentially abundant metabolite	OR (95% CI)^a^		FDR *P*
Citrate	14.54 (4.26–56.35)		0.04
Cholesterol	0.01 (0.001–0.10)		0.14
UM118961	0.52 (0.37–0.72)		0.11
UM210714	3.67 (1.93–7.33)		0.11

Pathway	Pathway impact factor	Hits	FDR *P*
Arginine biosynthesis	0.13	3	0.02
Glyoxylate and dicarboxylate metabolism	0.23	5	0.005
Citrate (TCA) cycle	0.14	3	0.04
Alanine, aspartate and glutamate metabolism	0.28	4	0.01
Aminoacyl-tRNA biosynthesis	0.17	5	0.01
Glycine, serine and threonine metabolism	0.28	3	0.14

^a^Odds ratio > 1 indicates association of higher abundance of metabolites with aoCRC versus yoCRC. The results are graphically represented in Figs. 1 and 2.

FDR, false discovery rate.

No significant differentially abundant metabolites were found between the following cohorts: patients with eoCRC vs. young non-CRC controls, patients with aoCRC vs. older non-CRC controls, and control subgroups (age < 50 vs. age > 60) (FDR *P* > 0.20 all).

The following differentially abundant metabolites were observed in the unadjusted comparison between patients with CRC and non-CRC controls (OR > 1 indicates greater abundance in CRC): UM41873 (OR 0.60, 95% CI 0.45–0.75, FDR *P* = 0.06) and 4-hydroxyhippuric acid (OR 0.45, 95% CI 0.30–0.67, FDR *P* = 0.09) in the unadjusted analyses. These differences were not significant after adjustment for the following covariates—sex, race, hyperlipidemia, obesity, cancer stage, and primary tumor location (FDR *P* = 0.71 and 0.49, respectively).

The heatmap of all metabolites analyzed (including those not statistically significant) is shown in Supplementary Fig. 1 and individual profiles of metabolites with *P* < 0.05 are shown in Supplementary Fig. 2. Only nine metabolites were differentially abundant in both eoCRC vs. aoCRC and age < 50 non-CRC controls vs. age > 60 non-CRC controls.

### Pathway analysis results

**Figure 2:**
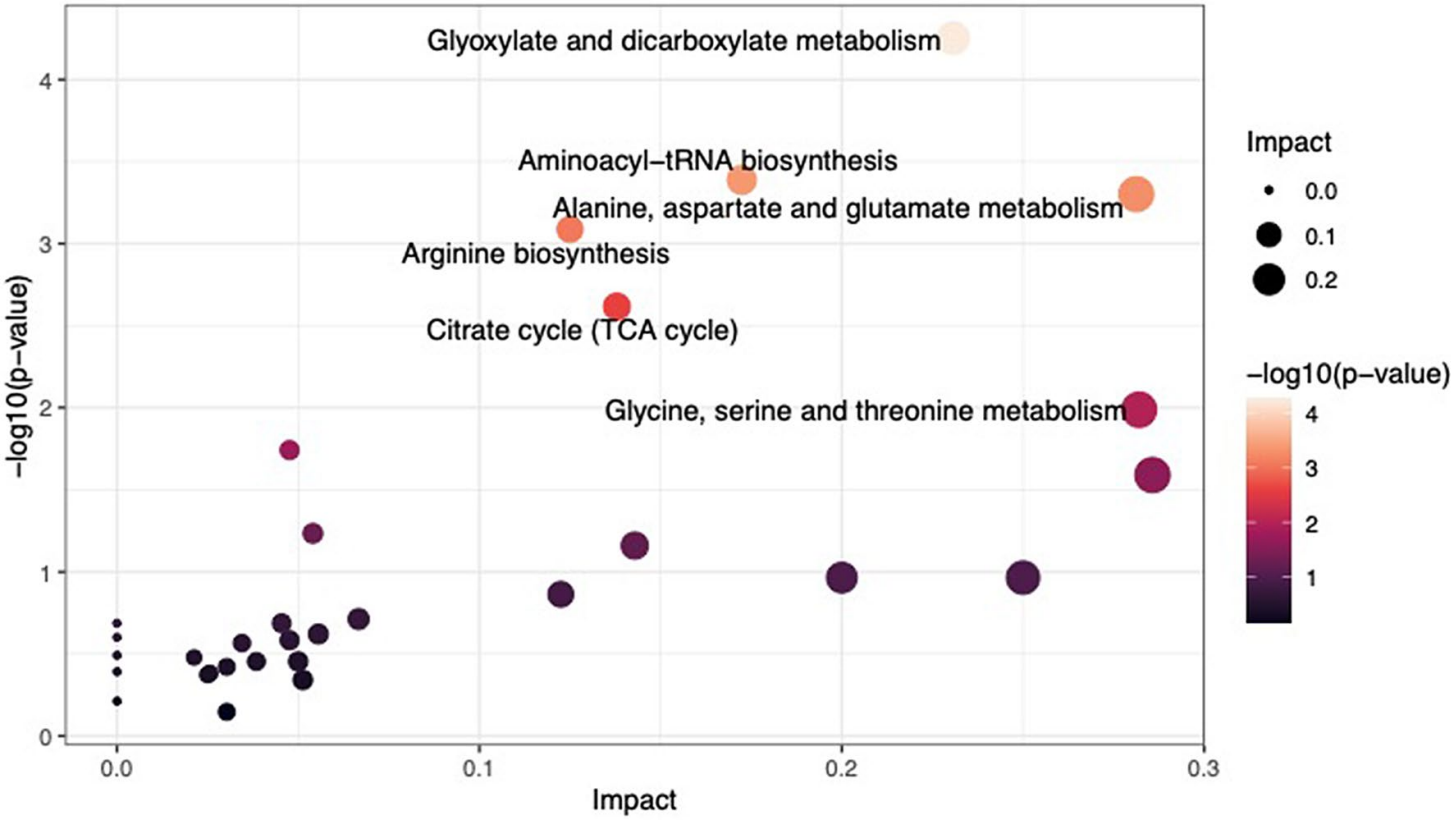
Bubble plot of pathway impact analyses. The impact factor topology analysis measures the impact of each metabolite in a pathway on a set of other metabolites in a pathway. Topology analysis was performed on the differentially expressed metabolites to estimate their cumulative impact on the pathways, corresponding values are described in Table 1.

**Figure 3:**
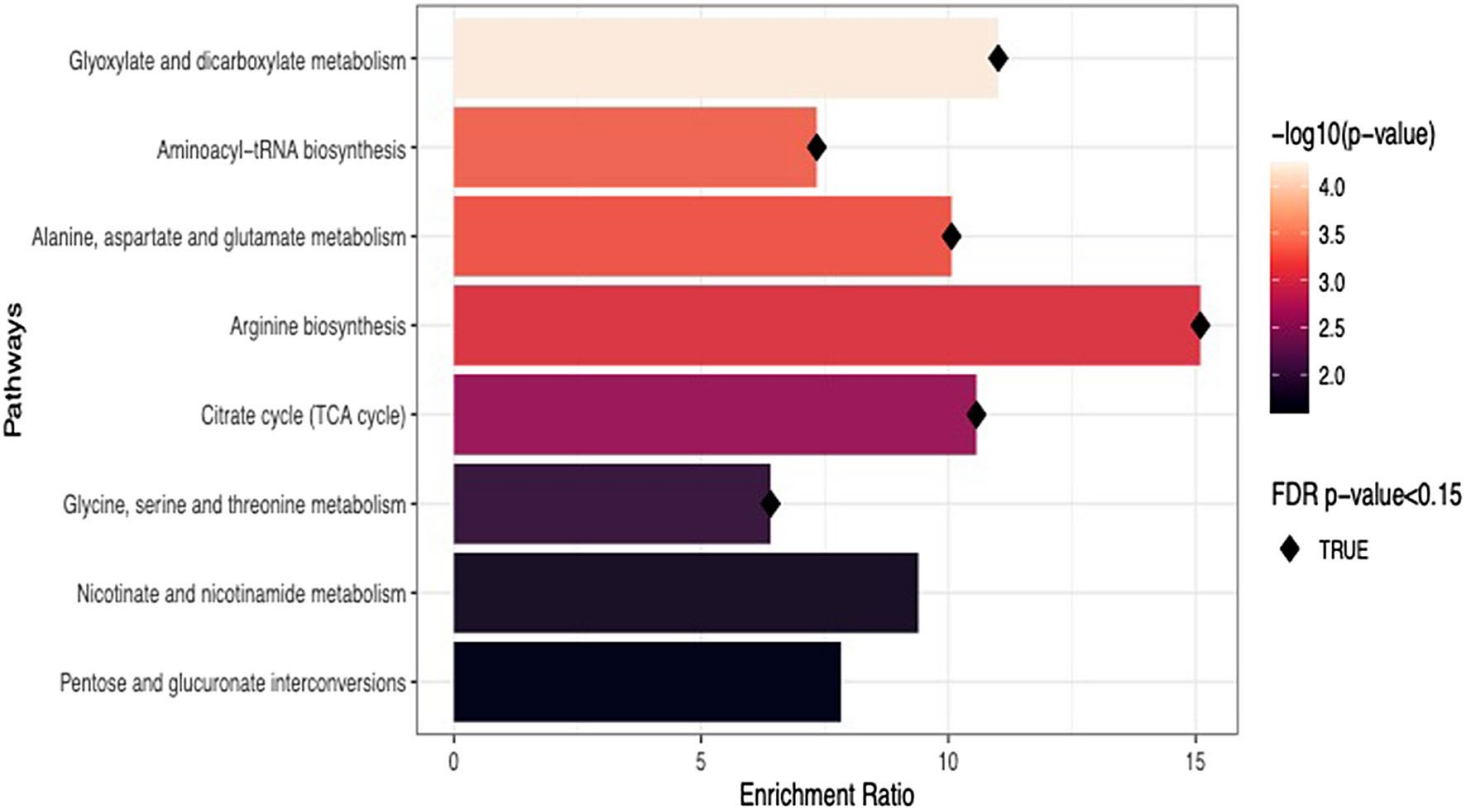
Results for pathway enrichment analyses. Enrichment analyses test whether more significant metabolites are involved in a pathway than expected by chance. The metabolites involved in the pathways are shown in the next figure (Fig. 4). FDR, false discovery rate.

**Figure 4:**
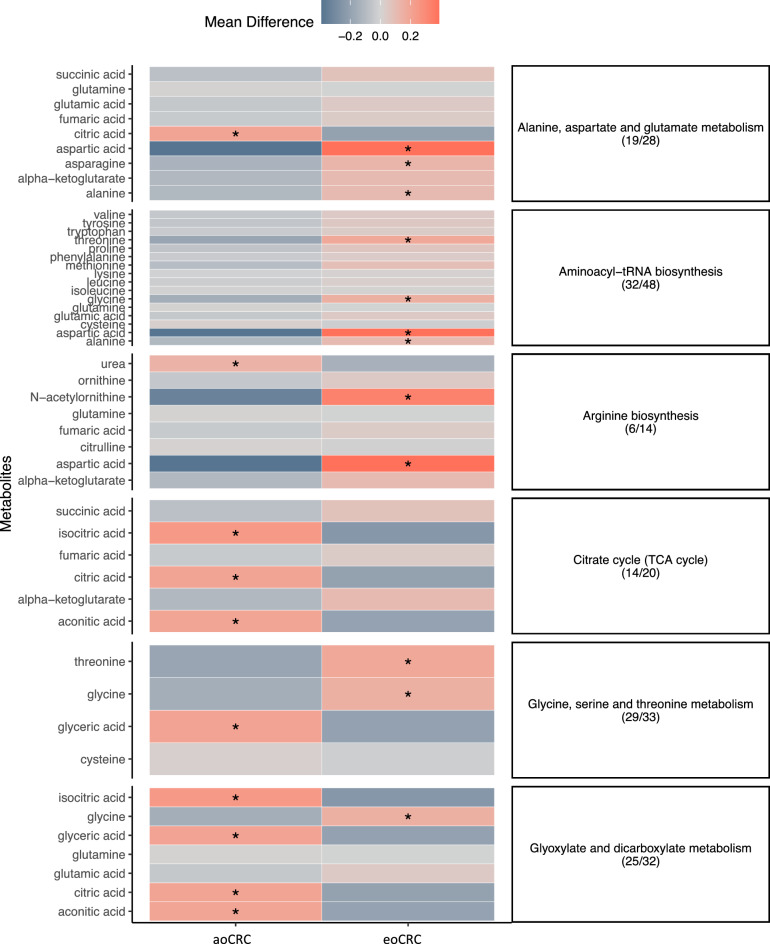
Heatmap outlining the metabolites significantly upregulated or downregulated in patients with eoCRC and aoCRC impacting the corresponding pathways. The heatmap was generated using R package ggplot2^49^, *Indicate significantly altered metabolites. The numbers within parenthesis indicates the fraction of samples that exhibit the specific altered metabolite. eoCRC, early-onset colorectal cancer; aoCRC, average-onset colorectal cancer.

**Figure 5:**
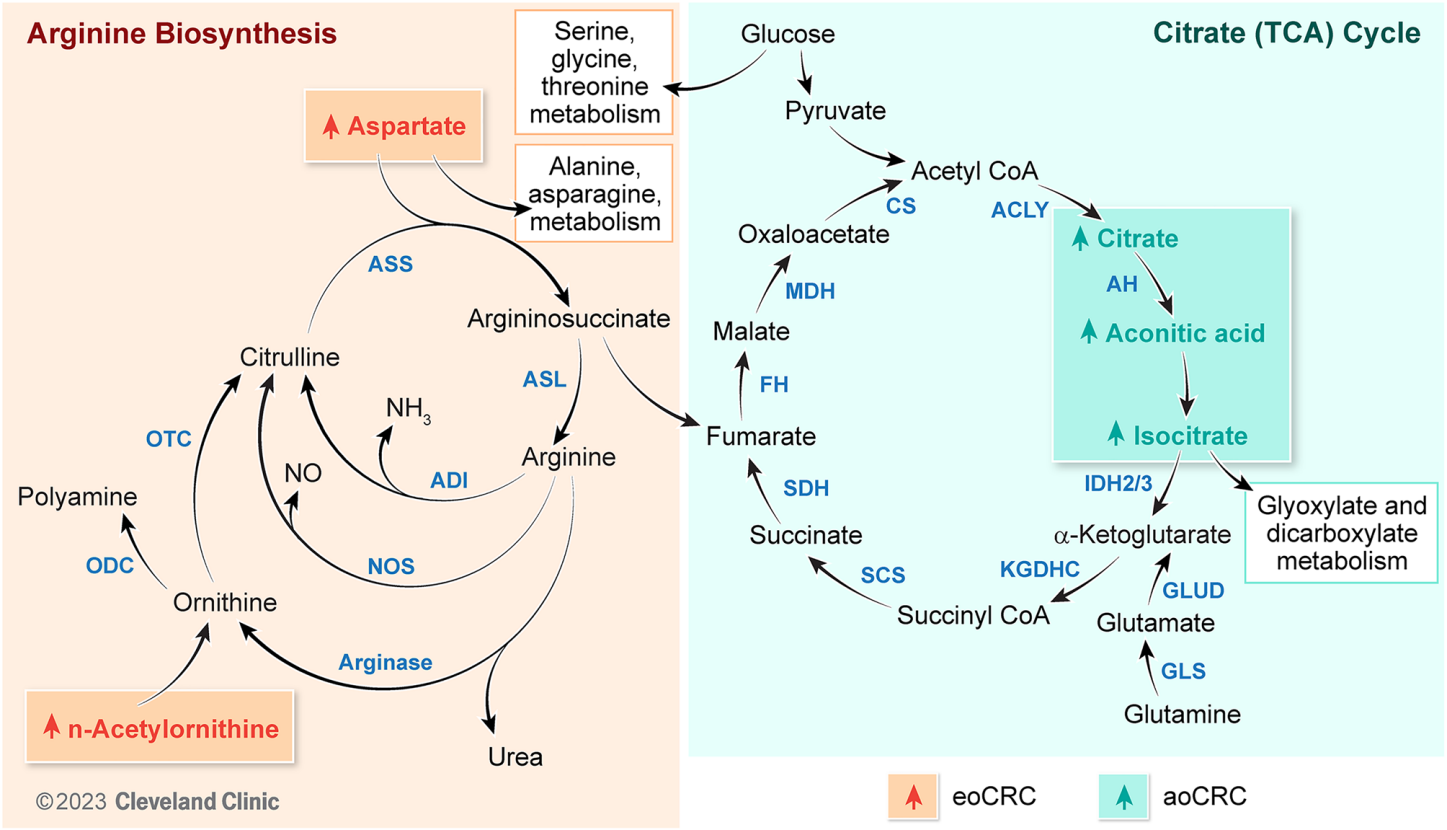
Illustration mapping the metabolic alterations in eoCRC and aoCRC to the interlinked arginine biosynthesis and TCA cycles. Arginine biosynthesis: ADI, arginine deiminase; ASL, argininosuccinate lyase, ASS, argininosuccinate synthetase; NOS, nitric oxide synthase, NO, nitric oxide, ODC, ornithine decarboxylase, OTC, ornithine transcarbamylase**.** Citrate (TCA) Cycle: CS, citrate synthase, ACLY, adenosine triphosphate citrate lyase, AH, aconitase, IDH, isocitrate dehydrogenase, KGDHC, α-ketoglutarate dehydrogenase complex, SCS, succinyl-CoA synthase; SDH, succinate dehydrogenase; FH, fumarate hydratase; MDH, malate dehydrogenase; GLS, glutaminase; GLUD, glutamate dehydrogenase**.** eoCRC, Early-Onset Colorectal Cancer; aoCRC, average-onset colorectal cancer.

Metabolic pathways impacted by the differentially abundant metabolites included: carbohydrate metabolism (citrate/TCA cycle, FDR *P* = 0.04, impact factor = 0.14), carbohydrate biosynthesis (glyoxylate and dicarboxylate metabolism, FDR *P* = 0.005, impact factor = 0.23), amino acid metabolism (alanine, aspartate, and glutamate metabolism, FDR *P* = 0.01, impact factor = 0.28; glycine, serine, and threonine metabolism, FDR *P* = 0.14, impact factor = 0.28; arginine biosynthesis, FDR *P* = 0.02, impact factor = 0.13, and amino-acid t-RNA biosynthesis, FDR *P* = 0.01, impact factor = 0.17). The results are summarized in Table 2 and graphically represented in Fig. 2. There were no significant metabolomic differences between the young and older non-CRC controls. The arginine biosynthesis pathway, followed by the glyoxylate and dicarboxylate metabolism and TCA cycle pathways, were the most enriched with differentially expressed metabolites (Fig. 3). The Fig. 4 heatmap demonstrates the individual metabolite alterations in the pairwise comparison of patients with eoCRC and aoCRC reflected in the pathway analyses. The metabolic pathways with upregulated metabolites in eoCRC included: alanine, aspartate, and glutamate metabolism; aminoacyl-tRNA biosynthesis; arginine biosynthesis; glycine, serine, and threonine metabolism. The metabolites upregulated in multiple pathways included aspartic acid, alanine, glycine, and threonine. The pathways with upregulated metabolites in aoCRC (citric acid, isocitric acid, acotinic acid) were the TCA cycle and glyoxylate and dicarboxylate metabolism. These metabolite level changes were mapped to the interlinked metabolic pathways as represented in Fig. 5.

### Survival analyses

Overall survival (OS) analysis was performed in patients with CRC. The median follow-up period was 42 months (IQR = 26–64 months) for patients with eoCRC and 44 months (IQR = 29–84 months) for patients with aoCRC. During follow-up, 85 patients died (34.8% with eoCRC and 59.6% with aoCRC, P = 0.003).

Supplementary Fig. 3 summarizes the results of adjusted and unadjusted Cox regression analyses outlining both significant and non-significant results. In adjusted analyses, 4-hydroxyhippuric acid was significantly associated with OS in patients with CRC (HR = 0.4, 95% CI 0.3–0.7, FDR *P* = 0.05). As shown in Supplementary Fig. 4, the 25th percentile of 4-hydroxyhippuric acid abundance was associated with worse OS vs. the 75th percentile (45.98% vs. 62.16%). It was not associated with OS in patients with either eoCRC or aoCRC separately.

Adipic acid was statistically significantly associated with OS in patients with aoCRC in the unadjusted analysis (HR = 3.1, 95% CI 1.7–5.6, FDR *P* = 0.13). Compared with the 25th percentile of adipic acid abundance, the 75th percentile was associated with a lower 60-month OS in patients with aoCRC (47.15 vs. 62.91%) (Supplementary Fig. 5). However, this association was no longer significant in the adjusted analysis (HR = 2.6, 95% CI 1.2–5.5, FDR *P* = 1). Adipic acid was not associated with survival in patients with eoCRC or when eoCRC and aoCRC were combined.

## Discussion

We identified significant differences in metabolites and associated pathways in comparing patients with eoCRC and aoCRC along with age-appropriate non-CRC controls. In particular, higher citrate levels were associated with aoCRC compared to eoCRC with an odds ratio of 21.8 (95% CI 5.0–110.9.35, FDR *P* = 0.04) on adjusted analyses. The arginine biosynthesis pathway, followed by the glyoxylate and dicarboxylate metabolism pathways and TCA cycle, were the most enriched, based on the relative abundance of metabolites in this study. Other pathway alterations reflected in metabolite levels and captured by metabolomics included the aminoacyl t-RNA biosynthesis; alanine, aspartate, and glutamate metabolism; and glycine, serine, and threonine metabolism. Similar studies comparing patients with colorectal cancer to those without cancer have identified additional pathway differences, such as lipid metabolism, which may be associated with variations in technique or patient selection^11,12^. Interestingly, two unidentified metabolites (UM118961 and UM210714) were observed and will require further exploration in future studies^27,28^. Association of higher levels of 4-hydroxyhippuric acid with improved overall survival was observed in the whole cohort.

Recent research has demonstrated the ability of metabolomics to identify metabolic pathway alterations associated with CRC^11,29–37^. These include TCA cycle-related metabolites reflecting impaired mitochondrial respiration and oxidative stress^10,11,32^. The Warburg effect, which involves increased aerobic glycolysis in cancer cells, also impacts the levels of the related TCA cycle metabolites^7,29–31,38,39^. It is noteworthy that the alterations in these pathways are variable depending on the age of onset for CRC. In the present study, the TCA cycle metabolites including the key metabolite citrate were more abundant in aoCRC. As citrate has been proposed as a biomarker in gastrointestinal cancers, further investigations are necessary to understand the differences between eoCRC and aoCRC and their clinical significance^39,40^. These findings are relevant from a therapeutic perspective, as there are ongoing efforts to explore options for targeting the TCA cycle in GI cancers^41,42^.

In the present study, patients with eoCRC exhibited enrichment with differentially expressed metabolites in the arginine biosynthesis pathway. Arginine serves a crucial function in tumor metabolism, including the synthesis of nitric oxide, polyamines, proline, and glutamate^43^. Dietary correlations have also been made between red meat consumption and polyamine synthesis impacting colorectal cancer risk^43^. Various strategies are being explored to target this pathway, including arginine deprivation, arginine uptake inhibition, and micro RNA-mediated modulation of the enzymes involved^43^. Investigations are also ongoing to inhibit Arginosuccinate synthetase 1(ASS1), a key enzyme involved in the arginine biosynthesis pathway that is upregulated in colon cancer^44^. Upregulation of ASS1 enhances the ability of cells to recycle arginine and be less vulnerable to arginine deprivation, and inhibition of ASS1 has been shown to successfully impair the pathogenicity of colon cancer cells^44^^,^^43^. Further enhancement of this synthetic lethality is possible by arginine deprivation and inhibition of any escape pathways utilized by the cells for survival^45,46^. The mapping of the metabolic pathway for arginine cycle metabolites in eoCRC is comparable to the effects observed with ASS1 inhibition and arginine deficiency. This results in high levels of aspartic acid, asparagine, and ornithine, as well as lower levels of TCA cycle metabolites and increased levels of serine biosynthesis cycle metabolites^46,47^. These unique metabolic characteristics in arginine biosynthesis, as opposed to aoCRC, provide potential avenues for innovative therapeutic targeting.

Unique associations between metabolites and overall survival were noted when stratified by age. Hippuric acid is an important energy metabolism precursor, and lower levels of hippuric acid are noted in cancer subjects, likely due to increased consumption by cancer cells^48^. However, definite conclusions cannot be drawn from these results, particularly due to the sample size of patients in our study.

A few limitations of the study are worth noting. Firstly, the small sample size of the non-CRC control group may have prevented us from identifying key metabolic differences between patients with CRC and non-CRC controls. However, we had sufficient patients with CRC to observe statistically significant differences in metabolites with an exploratory threshold, and the limitations of the control group did not restrict our ability to detect meaningful differences between early and average onset CRC. Moreover, if we assume that the effect sizes are due to age only and remain within the 95% confidence interval regardless of CRC status, we had at least 80% power to be able to detect the differences between healthy older adults and healthy younger adults for the metabolites that were significantly altered in patients with CRC (citrate and cholesterol). . While the point estimate of 21.8 associated with the odds ratio (OR) for citrate suggests a strong association, the wide confidence interval could reflect some uncertainty surrounding the estimate, likely due to the small sample size. This highlights the importance of further validation of these findings in a larger study. Secondly, selection bias may have influenced baseline differences in comorbidities between patients with CRC and non-CRC controls. To address this, we included comorbidities as covariates in our regression models. Thirdly, given that an individual's exposure is variable and dynamic throughout their lifetime, confounding factors such as dietary and medication exposures could certainly influence the results. Chemotherapy administration could also affect the metabolome. In our study, only those with stage IV colorectal cancer were receiving chemotherapy near the time of sample collection. While we did not directly adjust for chemotherapy administration, we did adjust for the stage of disease, which is a proxy for the effect of chemotherapy administration. The adjustment of stage is also expected to address the differences in the proportions of patients with stage IV cancer including colorectal liver metastases that may impact the metabolome. We ensured uniformity in the sample collection timing as all blood samples were collected on the day of the procedure and after overnight fasting. Finally, we adjusted for primary tumor sidedness to address the confounding effect of different molecular alterations in left versus right-sided CRC, but residual confounding is still possible. Since all stage IV patients in this study had resectable liver metastases, it is also unclear if these results can be generalized to other metastatic sites such as lung or peritoneum.

Accounting for the heterogeneous molecular alterations presents inherent challenges from a combination of factors. First, the majority of the patient cohort under investigation had early-stage disease, rendering molecular profiling data unavailable as it is not a routine standard of care in such cases. Consequently, stratifying the cohort based on diverse molecular characteristics would have resulted in the creation of numerous subgroups and compromised the statistical power of the study in discerning metabolomic differences, which was the primary research aim. Moreover, the observed molecular differences did not exhibit substantial numerical differences between eoCRC aoCRC.

Key findings of the study include the significant differential abundance of citrate in aoCRC compared to eoCRC with an odds ratio of 21.8 in adjusted analysis. Further investigations related to mechanisms underlying metabolomic differences in arginine biosynthesis and potential relationships with environmental exposures may elucidate the pathogenesis of eoCRC and offer opportunities for therapeutic targeting. We observed the association of 4-hydroxy Hippuric acid with overall survival, which could serve as a prognostic biomarker. In addition to further validation with targeted metabolomics, future efforts should include correlation with metagenomics and mechanistic studies.

## Data and materials availability

TJ and SK had full access to all data in the study. We take full responsibility for the integrity of our data. The data generated in this study are not publicly available because the information could compromise patient privacy and consent, but are available upon reasonable request from the corresponding author.

### Supplementary Information

Supplementary Information.
