# Supplementary material for: Plasma metabolomic differences in early-onset compared to average-onset colorectal cancer
Source: Sci Rep. 2024 Feb 21;14:4294. doi: 10.1038/s41598-024-54560-5 (PMC10881959; doi:10.1038/s41598-024-54560-5)
Supplement: Supplementary file 1 — Supplementary Information. [file 41598_2024_54560_MOESM1_ESM.docx]

**Supplementary Figures and Table:**

**Supplementary Figure 1.** Heatmap of all metabolites analyzed*^a^

*The heatmap shows the abundance of all metabolites. Age, sex, and cancer stages are annotated at the top. Agglomerative hierarchical clustering with complete linkage and Euclidean distance measure was used to cluster groups and patients within groups.

^a^ The heatmap was generated using used R package ComplexHeatmap (Gu, Z. (2016) Complex heatmaps reveal patterns and correlations in multidimensional genomic data. Bioinformatics; Gu, Z. (2022) Complex Heatmap Visualization. iMeta)

**Supplementary Figure 2.** Heatmap of metabolites with *P*<0.05^a^

^a^ The heatmap was generated using used R package ComplexHeatmap (Gu, Z. (2016) Complex heatmaps reveal patterns and correlations in multidimensional genomic data. Bioinformatics; Gu, Z. (2022) Complex Heatmap Visualization. iMeta)

**Supplementary Figure 3.** Graphical representation of cox regression analysis. All the metabolites with a *P*-Value<0.5 are included in the figure. The metabolites represented in orange colour were statistically significant by the FDR Cut-off <0.15.

*Metabolites of relevance (significant by FDR P cut-off in any one cohort), included in the results section and discussion.

**Supplementary Figure 4.** Kaplan-Meir survival curve demonstrating the relationship of 4-hydroxyhippuric acid levels with survival in whole cohort of CRC

**Supplementary Figure 5.** Kaplan-Meir survival curve demonstrating the relationship of adipic acid levels with survival in aoCRC

**Supplementary Table 1:** Results for MSI status and somatic mutations for patients included in the study

| Characteristics | All CRC (n=170) |  | eoCRC (n=66) |  | aoCRC (n=104) |  |
| --- | --- | --- | --- | --- | --- | --- |
|  | n | % | n | % | n | % |
| MSI-H | 5 | 2.94 | 1 | 1.52 | 4 | 3.85 |
| MSI-S | 107 | 62.94 | 49 | 74.24 | 58 | 55.77 |
| MSI-Unknown | 58 | 34.12 | 16 | 24.24 | 42 | 40.38 |
|  |  |  |  |  |  |  |
| KRAS mutation |  |  |  |  |  |  |
| Present | 42 | 24.71 | 19 | 28.79 | 23 | 22.12 |
| Absent | 54 | 31.76 | 28 | 42.42 | 26 | 25.00 |
| Unknown | 74 | 43.53 | 19 | 28.79 | 55 | 52.88 |
|  |  |  |  |  |  |  |
| NRAS mutation |  |  |  |  |  |  |
| Present | 1 | 0.59 | 1 | 1.52 | 0 | 0.00 |
| Absent | 69 | 40.59 | 38 | 57.58 | 31 | 29.81 |
| Unknown | 100 | 58.82 | 27 | 40.91 | 73 | 70.19 |
|  |  |  |  |  |  |  |
| BRAF mutation |  |  |  |  |  |  |
| Present | 7 | 4.12 | 5 | 7.58 | 2 | 1.92 |
| Absent | 59 | 34.71 | 32 | 48.48 | 27 | 25.96 |
| Unknown | 104 | 61.18 | 29 | 43.94 | 75 | 72.12 |
|  |  |  |  |  |  |  |
| Her2 amplification |  |  |  |  |  |  |
| Present | 3 | 1.76 | 1 | 1.52 | 2 | 1.92 |
| Absent | 14 | 8.24 | 8 | 12.12 | 6 | 5.77 |
| Unknown | 153 | 90.00 | 57 | 86.36 | 96 | 92.31 |

MSI-H: Microsatellite instability- high

MSS: Microsatellite stable
